# Magnetic resonance imaging of bacterial and tuberculous spondylodiscitis with associated complications and non-infectious spinal pathology mimicking infections: a pictorial review

**DOI:** 10.1186/s12891-017-1608-z

**Published:** 2017-06-05

**Authors:** Yogesh Kumar, Nishant Gupta, Avneesh Chhabra, Takeshi Fukuda, Neetu Soni, Daichi Hayashi

**Affiliations:** 1 Department of Radiology, Yale New Haven Health System at Bridgeport Hospital, 267 Grant Street, Bridgeport, 06610 CT USA; 2 Department of Radiology, St. Vincent’s Medical Center, 2800 Main Street, Bridgeport, 06606 CT USA; 3 Department of Radiology, University of Texas Southwestern Medical Center, 5323 Harry Hines Blvd, Dallas, 75390 TX USA; 4 Department of Radiology, The Jikei University School of Medicine, 3-25-8 Nishi-shimbashi, Minato-ku, Tokyo, 105-8461 Japan; 5 Department of Neuroradiology, Sanjay Gandhi Postgraduate Institute of Medical Sciences, Lucknow, U.P India; 6 Department of Radiology, Boston University School of Medicine, 820 Harrison Avenue, FGH Building 3rd Floor, Boston, 02118 MA USA

**Keywords:** Spine, Infection, Abscess, MRI, Spondylitis, Discitis

## Abstract

Magnetic resonance (MR) imaging plays an important role in the evaluation of bacterial and tuberculous spondylodiscitis and associated complications. Owing to its high sensitivity and specificity, it is a powerful diagnostic tool in the early diagnosis of ongoing infections, and thus provides help in prompt initiation of appropriate, therapy which may be medical or surgical, by defining the extent of involvement and detection of complications such as epidural and paraspinal abscesses. More specifically, MR imaging helps in differentiating bacterial from tuberculous infections and enables follow up of progression or resolution after appropriate treatment. However, other non-infectious pathology can demonstrate similar MR imaging appearances and one should be aware of these potential mimickers when interpreting MR images. Radiologists and other clinicians need to be aware of these potential mimics, which include such pathologies as Modic type I degenerative changes, trauma, metastatic disease and amyloidosis. In this pictorial review, we will describe and illustrate imaging findings of bacterial and tuberculous spondylodiscitis, their complications and non-infectious pathologies that mimic these spinal infections.

## Background

Magnetic resonance (MR) imaging plays an important role in the evaluation of bacterial and tuberculous spondylodiscitis. Owing to its high sensitivity and specificity of up to 90% or greater [1–5], it is a powerful diagnostic tool in the early diagnosis of spondylodiscitis. MR imaging thus provides help in prompt initiation of appropriate therapy which may be medical or surgical, by defining the extent of involvement and detection of complications such as epidural and paraspinal abscesses. However, other non-infectious pathology can demonstrate similar MR imaging appearances and one should be aware of these potential mimickers when interpreting MR images [6–10]. Radiologists and referring clinicians need to be aware of these potential mimics, which include such pathologies as Modic type I degenerative changes, trauma, metastatic disease and amyloidosis. In this pictorial review, we describe and illustrate imaging findings of bacterial and tuberculous spondylodiscitis, their complications and non-infectious pathologies that mimic these infections.

## Epidemiology of bacterial and tuberculous spondylodiscitis

In bacterial spondylodiscitis, *Staphylococcus aureus* is the most commonly responsible organism, accounting for up to >75% of cases [11–13]. Other organisms that may cause bacterial spondylodiscitis include *Escherichia coli* in patients with concurrent urinary tract infections, *Pseudomonas aeruginosa* in patients with a history of intravenous drug abuse, *Streptococcus pneumoniae* in patients with diabetes, and *Salmonella* species in patients with sickle cell disease or asplenia [14, 15].

Tuberculosis (TB) is one of the major causes of morbidity and mortality in developing countries with a rising trend in the United States and other developed countries with a reported worldwide incidence of 10.4 million cases in 2015 according to the World Health Organization global report [16]. Spinal TB is a destructive form of TB affecting the spinal column in less than 1% of all TB cases, causing neurological deficits, spinal deformities and paraplegia which mandates early diagnosis and treatment to avoid permanent damages [17]. According to an epidemiological study in the United States with a 10-year observation period (2002–2011), the incidence of spinal TB is actually decreasing from 0.07 cases per 100,000 persons in 2002 to 0.05 cases per 100,000 in 2011 (*p* < 0.001), corresponding to 1 case per 2 million persons in the latter year [18]. The same study identified men aged approximately 50 years were most commonly affected [18].

## Pathogenesis of bacterial and tuberculous spondylodiscitis

Bacterial spondylodiscitis usually occurs due to hematogenous spread from a distant site, particularly from the lung or urinary tract. Bacterial spondylodiscitis can also occur via direct extension, for instance from spinal surgery, myelography, penetrating trauma, and from adjacent infections in the thorax or abdomen. Due to differences in vascular anatomy at different stages of life, discs are usually the first site of infection in pediatric patients followed by metaphyseal involvement, while in adult patients endplates are usually the first site of infection followed by disc involvement [19, 20].

Tuberculous spondylodiscitis, also known as Pott disease, is caused by *Mycobacterium tuberculosis*, a slow-growing gram positive acid fast bacillus which becomes lodged in the bone via Batson’s venous plexus and lymphatic from primarily infected lung, lymph nodes, mediastinum and viscera, forming granulomatous inflammation and caseation necrosis [17]. Primary or secondary involvement of the posterior appendicular and articular element along with paraspinal soft tissue can also be seen [17, 21]. Thoracolumbar region is the most commonly affected site while the cervical and sacrum regions are less commonly involved. Usually more than one vertebra is affected because of its segmental arterial distribution and subligamentous spread of the disease. The bacilli reach the disc space causing disc destruction, spreads to adjacent vertebral bodies leading to vertebral collapse, anterior wedging, characteristic kyphotic angulation (Gibbus deformity), which may compress the spinal cord and nerve roots producing functional impairment [17, 22, 23].

## Clinical presentation and diagnostic work-up

Bacterial spondylodiscitis most commonly presents with acute to subacute onset of back pain and fever. In many cases, fever of unknown origin is the main presenting complaint. Progressively increasing pain at the surgical site is usually the first symptom of postsurgical infection. For tuberculous spondylitis, patients present with constitutional symptoms such as malaise, loss of weight and night sweats while in chronic healed stage patients present with back stiffness, deformity and neural deficits.

Laboratory evaluation includes elevated inflammatory markers such as erythrocyte sedimentation rate (ESR) and C-reactive protein (CRP). These are very sensitive but non-specific markers of active bacterial spondylodiscitis, and thus can only be used to exclude active infection. Moreover, normal levels can also be seen in chronic infections. Leukocytosis and positive blood cultures are seen in most cases, especially before the initiation of medical treatment. Diagnosis of TB infection is usually based on clinical features, cerebrospinal fluid (CSF) analysis, histology and culture. However, combined use of MR imaging and GeneXpert, a test which detects DNA sequences specific for *Mycobacterium tuberculosis* increases the sensitivity to 97.9% for detection [17]. In doubtful cases, tissue biopsy is required to reach the diagnosis.

The diagnosis is often made by a combination of clinical features and imaging findings in some cases of spinal infection to initiate early empirical treatment in order to reduce the risk of complications, such as vertebral collapse and cord compression. However, a definitive diagnosis of the causative micro-organism is very difficult to establish based on clinical features and imaging alone. Therefore, image-guided percutaneous spinal biopsies are being increasingly performed to get sufficient tissue for culture and sensitivity of the causative organism [24]. However, the biopsy diagnostic yield of percutaneous spinal biopsy for detecting infection ranges from 30 to 40% and aspiration of > or = 2 mL of purulent fluid reported increases the rate of positive cultures [24].

## Technical considerations for MR imaging

Usually, MR imaging of the spine is performed with basic sequences including T1 and T2 weighted sagittal and axial images. Additionally, fat-suppressed T2-weighted sequence or Short tau Inversion Recovery (STIR) sequence is used to increase the conspicuity of bone marrow edema and thus increasing the sensitivity [6]. If there is no bone marrow edema on fat suppressed T2-weighted or STIR images, gadolinium contrast administration does not add any value and is not required [25]. However, in many institutions, intravenous gadolinium contrast is usually administered in all suspected cases of vertebral infection. Its main role lies in differentiating phlegmon from epidural abscesses (this latter demonstrates peripheral enhancement with central non-enhancing component), which is very important in deciding the appropriate treatment as epidural abscesses require surgical treatment in many cases, while phlegmon is usually treated with medical treatment [26]. In patients who cannot undergo contrast-enhanced MRI due to contraindications such as poor renal function or allergic reactions, usefulness of diffusion weighted imaging (DWI) for detection of abscesses has been demonstrated in the literature [21, 27, 28]. Some authors advocate its routine use in clinical practice because DWI may help differentiate between abscess and other pathologies such as non-infected cystic lesions (postoperative seroma, hematoma), cerebrospinal fluid leak, cystic/necrotic tumor and unusual patterns of degenerative disc and facet joint changes [21]. For assessment of spinal and paraspinal abscesses, DWI is performed with b values of 50 and 1000 s mm^−2^ and the apparent diffusion coefficient (ADC) map [21]. Image distortion can be reduced by deploying the parallel imaging factor 2. A spin-echo-type echo planar DWI sequence which allows fast imaging is commonly used to reduce motion artifacts [21].

## MR imaging features of bacterial and tuberculous spondylodiscitis and associated complications

### Bacterial infection

**Fig. 1:**
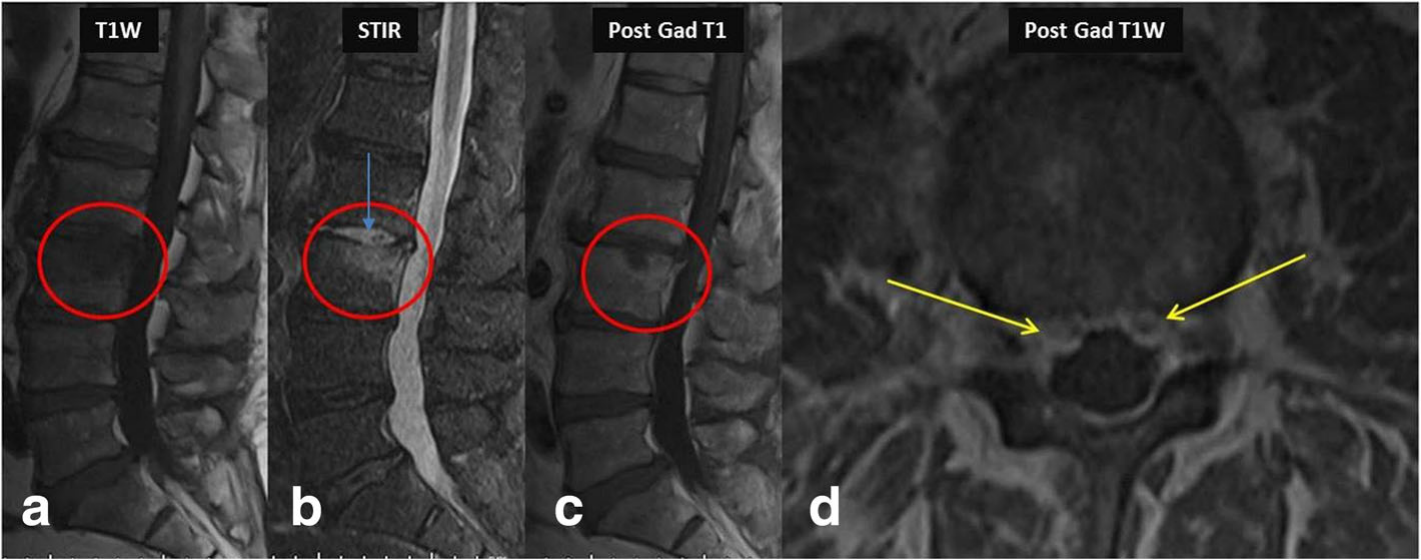
42-year-old-diabetic-male patient presented with low back pain for 2 weeks. MR imaging showed L2–3 vertebral endplate marrow edema (*red circle* in **a**, **b** and **c**) and increased fluid signal in the L2–3 intervertebral disc on STIR sequence (*blue arrow* in image **b**). Post contrast images (**c** & **d**) show enhancing soft tissue in the epidural space (*yellow arrows*), consistent with phlegmon, which is a very early complication. Post contrast imaging can help in differentiating between phlegmon, which does not contain non-enhancing or liquefied components, from an abscess which does have those components. *Streptococcus pneumoniae* was the causative organism

**Fig. 2:**
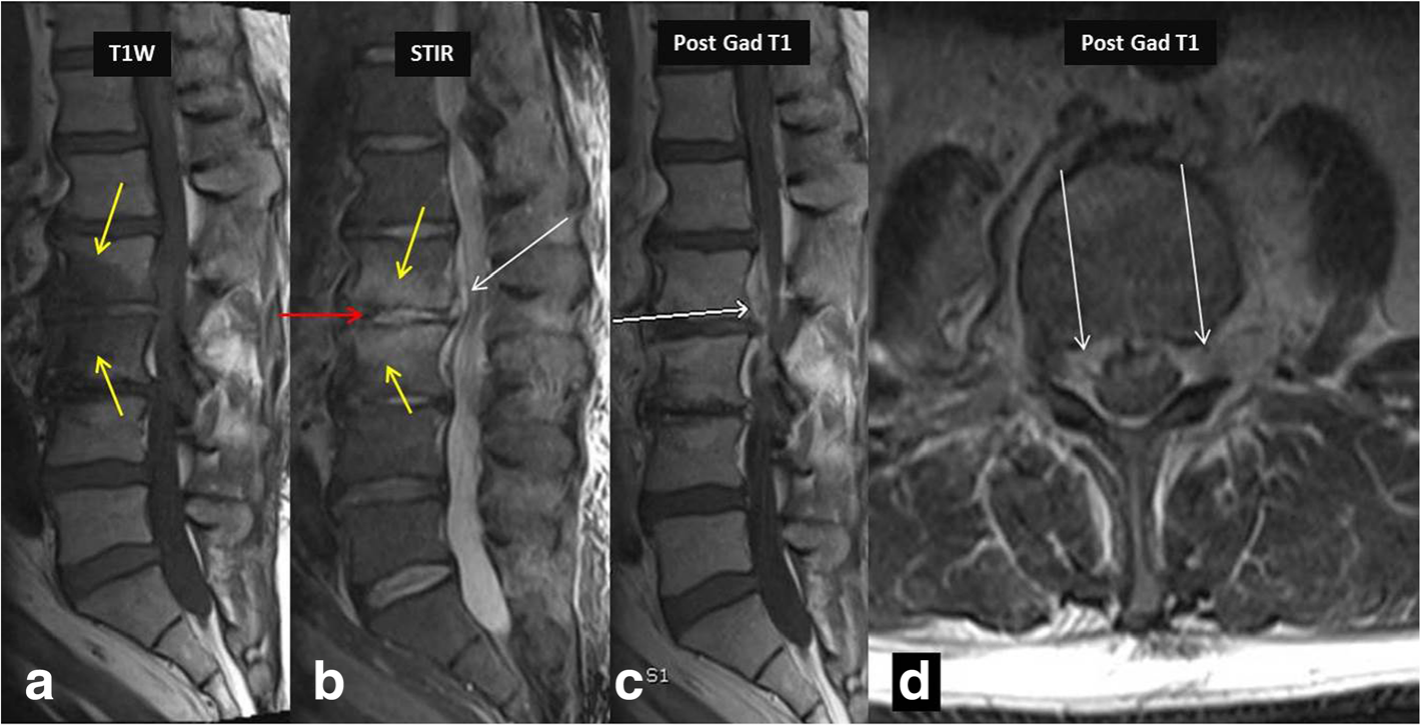
MR imaging in a 36-year-old intravenous drug abuser female shows marrow edema involving L2–3 vertebra reaching up to the endplates (*yellow arrows* in **a** and **b**), with minimal enhancement on post contrast imaging consistent with osteomyelitis. Note increased amount of fluid signal in L2–3 intervertebral disc suggesting discitis-osteomyelitis complex. Post contrast imaging shows enhancing epidural soft tissue (*white arrows* in **b**, **c** and **d**) without areas of liquefaction, consistent with epidural phlegmon. Once again post contrast imaging can differentiate epidural abscess from phlegmon and thus alter management. Also note the presence of left psoas muscle involvement. Responsible bacteria in this case were *Staphylococcus epidermidis*

**Fig. 3:**
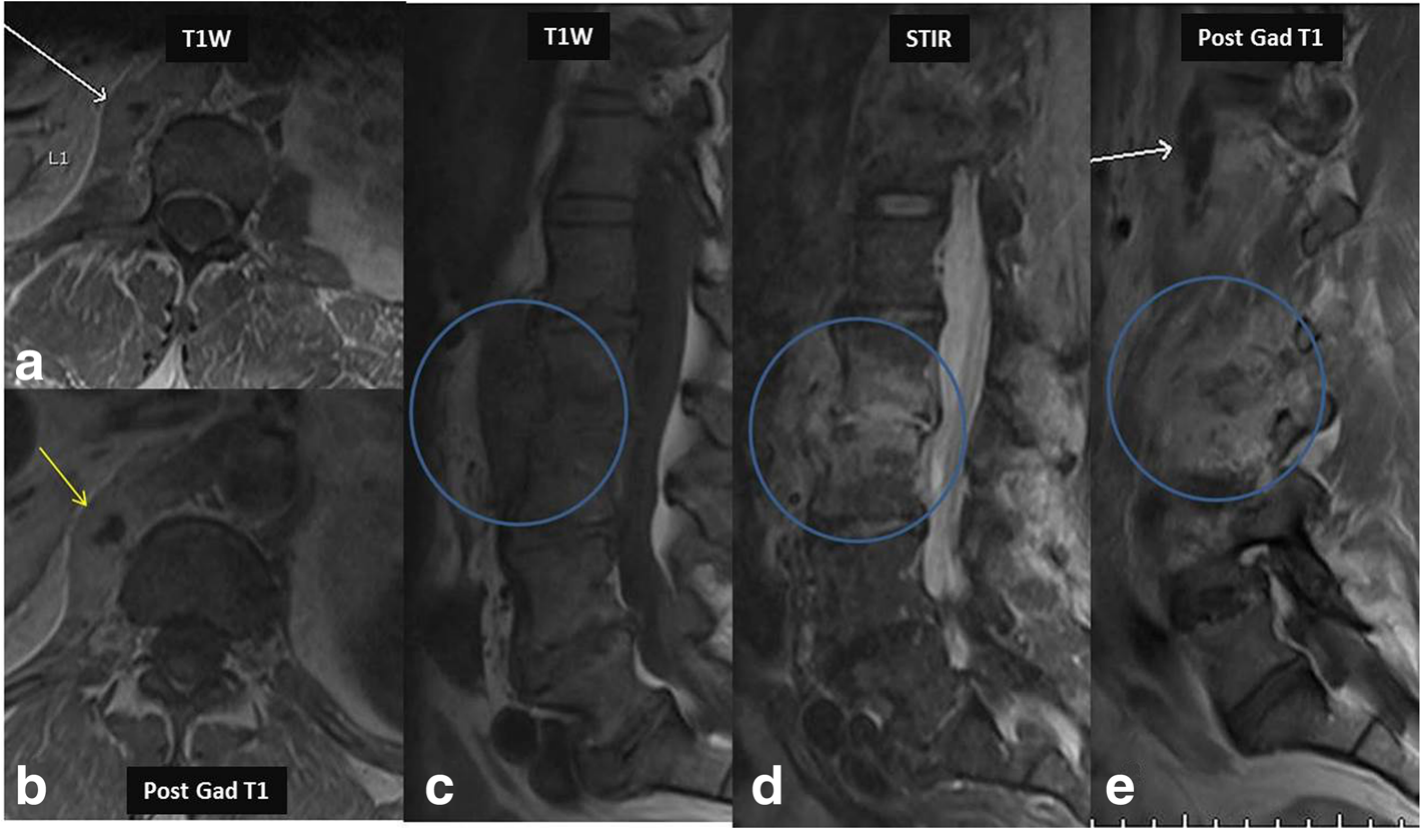
This is a companion case to the case shown in Fig. 2. 24-year-old male from India presented with low back pain. MR imaging showed involvement of the left diaphragmatic crura (*white arrow* in image **a**) and showing enhancement in post contrast imaging (*yellow arrow* in image **b**). Note the extensive prevertebral tubercular involvement (*blue circles* in **c**, **d** and **e**) on T1 W, STIR and post contrast imaging. *Staphylococcus aureus* was the causative organism

**Fig. 4:**
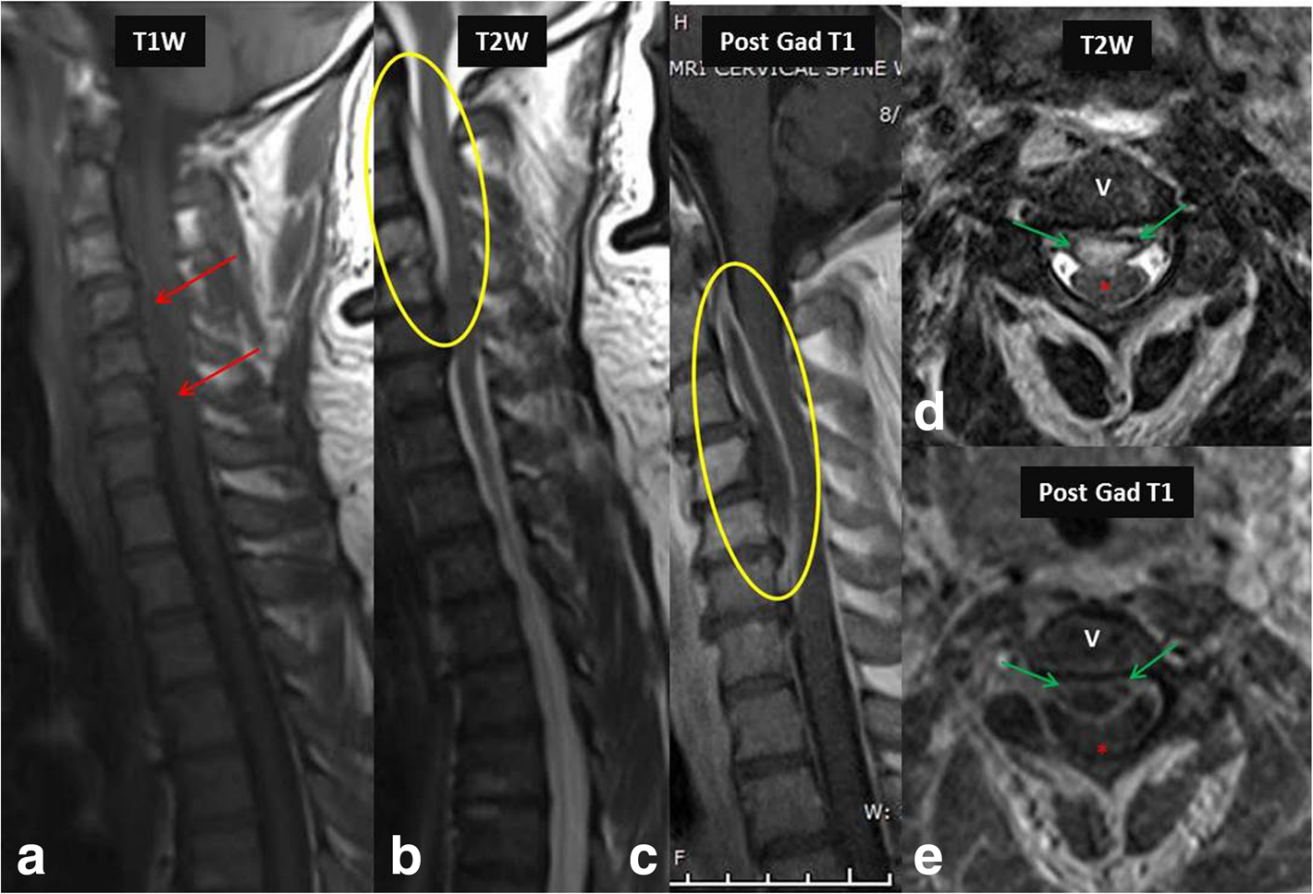
MR imaging in a 42-year-old female with paraparesis shows heterogeneous signal (*red arrows*) on T1 W image (**a**) and hyperintense epidural collection (*yellow oval*) on T2 W image (**b**) and show rim enhancement (*yellow oval*) on post contrast images (**c**), consistent with an epidural abscess. Axial T2 W (**d**) and post contrast images (**e**) distinctly show the epidural abscess (*green arrows*) causing indentation and posterior displacement of the thecal sac and cord (*red asterisk*). *Escherichia coli* was the causative organism. V- Vertebral body

**Fig. 5:**
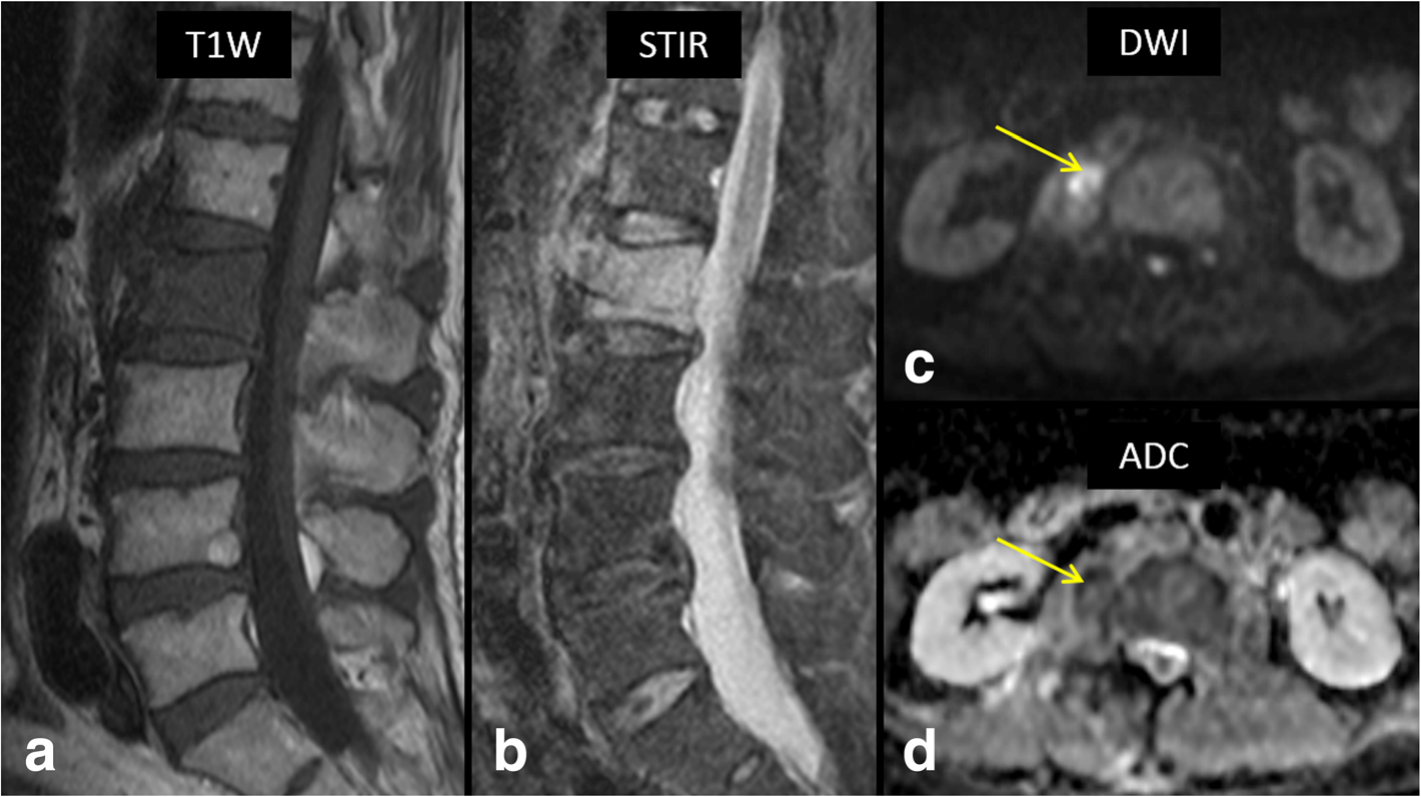
64-year-old male with fever and low back pain. Sagittal T1 W (**a**) and STIR (**b**) MR images show extensive bone marrow edema involving entire L2 vertebral body and anterior portion of L1 vertebral body. High b-value (1000) axial diffusion weighted image (**c**) shows a right paraspinal soft tissue lesion with restricted diffusion (*arrow*) with associated decreased ADC values on ADC map (**d**, *arrow*). Biopsy of the lesion confirmed bacterial spondylodiscitis at L1/2 with a paraspinal abscess. *Staphylococcus aureus* was the causative organism

Early bacterial infection within bone marrow typically results in minor ischemia due to septic embolism to the end arteries and the presence of inflammatory exudate. These changes result in low T1 and high T2 signal abnormalities in the bone marrow. High T2 signal intensity abnormalities within the bone marrow are made more conspicuous using fat-suppressed T2 weighted or STIR sequences. In addition, enhancement would be seen in the bone marrow (Fig. 1). The intervertebral disc will also show increased T2 signal with contrast enhancement. With progression of the disease without prompt and appropriate treatment, the end plates will show erosions with subsequent loss of disc and vertebral height. Pre- and paravertebral edema and contrast enhancement is also seen in most of the cases (Fig. 2). Extension into the adjacent soft tissue structures such as psoas muscle and diaphragmatic crura with phlegmon and abscess formation can also occur (Fig. 3). One of the major roles of MR imaging in evaluation of spinal infections is to look for spread of the infection in the epidural space and spinal canal with any effects on the cord and cauda equina nerve roots (Fig. 4). Contrast administration is necessary to differentiate phlegmon from the abscess in the epidural space (Figs. 2 and 4) as medical treatment may be sufficient in the former, while surgical drainage of abscess combined with medical treatment is necessary in cases of epidural abscesses. In patients who cannot receive gadolinium contrast or in cases where contrast enhanced MRI was not clearly diagnostic, DWI can be used to diagnose epidural abscess, as described earlier (Fig. 5).

**Fig. 6:**
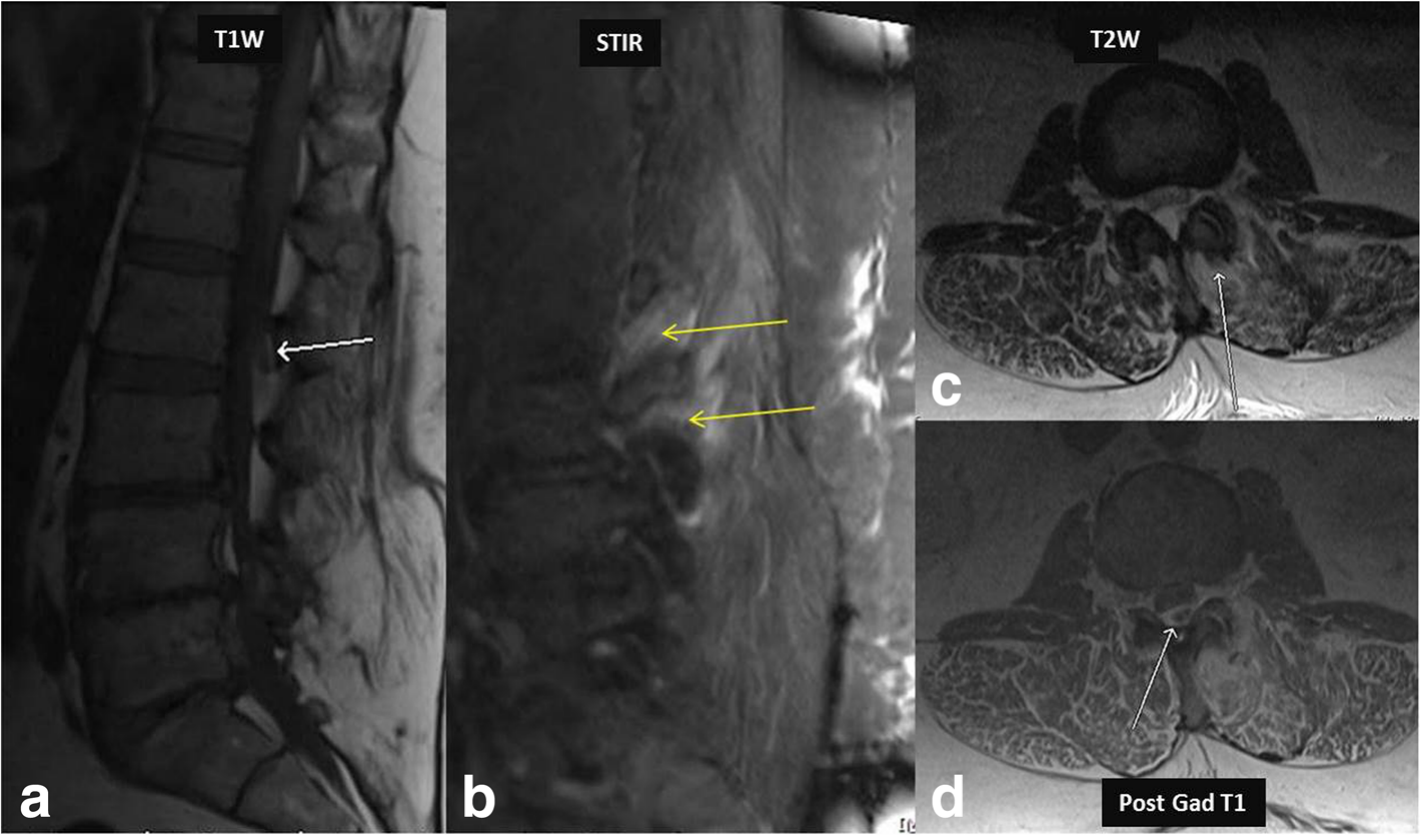
46-year-old-cashier presented with left lower back pain, especially with bending to the left for picking up shopping bags for customers. Physical exam showed left lower back point tenderness. T1 W images (**a**) showed a left sided hypointense posterior epidural lesion (*white arrow* in image **a**). STIR image (**b**) shows extensive edema in the region of left L2–3 lumbar facet joint (*yellow arrows*). T2 W image (**c**) shows fluid signal in left L2–3 facet joint with adjacent edema (*white arrow* in image **c**). Note the associated posterior epidural abscess (*white arrow* in image **d**) causing anterior displacement of the thecal sac. *Staphylococcus aureus* was the causative organism

**Fig. 7:**
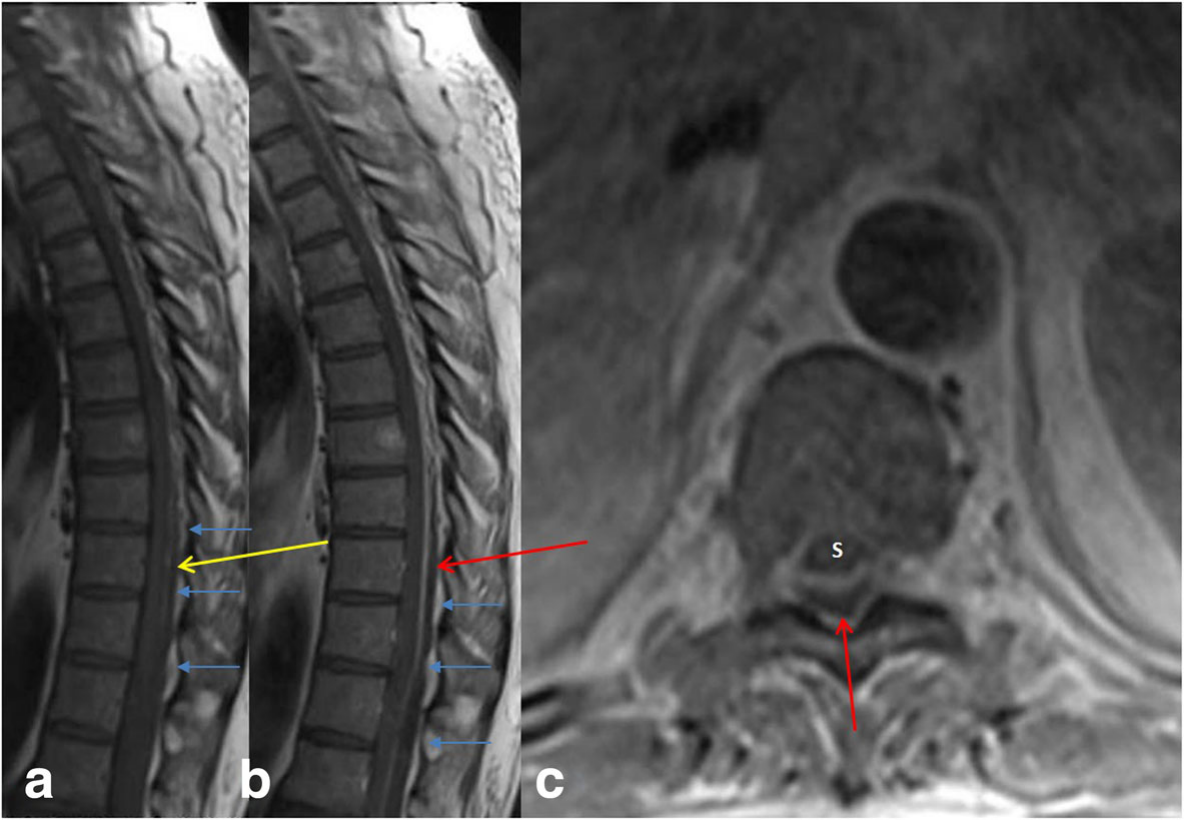
MR imaging in a 24-year-old intravenous drug abuser male shows heterogeneous signal in the subdural space in the lower thoracic spine (*yellow arrow* in **a**) on T1 W images. There is peripheral rim enhancement on post contrast T1 W images (*red arrows* in **b** and **c**), consistent with subdural abscess causing anterior displacement of the cord (marked as S in **c**). Also note the preserved epidural fat suggesting subdural location of the abscess (*blue* arrows in **a** and **b**). Biopsy of the abscess confirmed *Pseudomonas aeruginosa* infection

In some cases, such as elderly patients, immunocompromised patients and following spinal procedures including facet joint steroid injections, facet joints can be the primary site of infection. This can result in abscesses which tend to be located in the posterior epidural space as compared to anterior epidural space in cases of spondylodiscitis. The MRI findings of facet joint infection includes increased signal in the joint and surrounding edema (Fig. 6). These changes can be very subtle and resemble degenerative joint disease. However, clinical presentation suggesting infective etiology and the presence of an epidural abscess favors infection over degenerative changes. In some cases, subdural abscess may be the main presentation of the spine infections, which is seen as a collection deep to the epidural fat (Fig. 7).

DWI has been shown to be useful in highlighting the extent of bacterial spondylodiscitis and distinguishing it from other pathologies which can mimic it [29]. ‘Claw sign’, described as a well-marginated, linear region of restricted diffusion at the interface of normal and abnormal marrow within the adjacent vertebral bodies, is seen in Modic type I degenerative changes and can be used to differentiate it from infection. This is due to the fact that a gradual process such as degenerative disc disease is expected to produce a well-defined response while infections usually progress quickly and diffusely giving rise to diffuse diffusion abnormalities rather than well-defined linear diffusion changes. DWI is also helpful in differentiating normal postsurgical fluid collections from the infected collections as the latter would show restricted diffusion due to thick viscous pus (Fig. 5) [30].

Frank bony and disc destruction is usually seen in chronic cases when there is a lack of initiation of prompt and appropriate treatment, either due to delay in presentation or diagnosis. Reactive bony changes, such as new bone formation and sclerosis, normalization of T1 signal intensity within a previously infected vertebral body and resolution of bone marrow edema on T2 weighted images suggest bone healing in a resolving infection.

### Tuberculous spondylitis

**Fig. 8:**
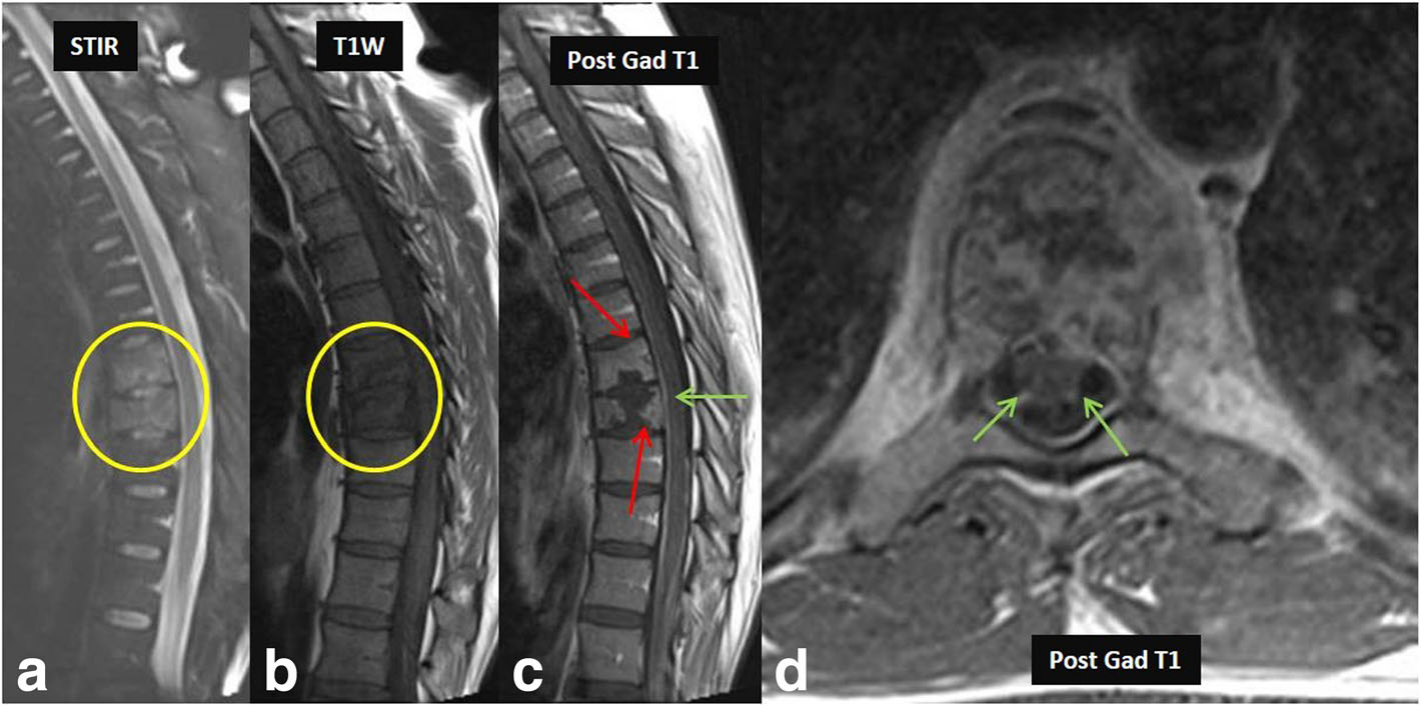
27-year-old-male patient who was an immigrant from South America presented with back pain. Initial MRI was normal. Follow up MRI of the thoracolumbar spine for persistent back pain revealed marrow edema in the lower thoracic spine (*yellow circle* in **a** and **b**). Post contrast imaging showed discitis-osteomyelitis complex (*red arrows* in **c**). Note the enhancement of nerve roots suggesting extension of infectious process to the nerve roots causing arachnoiditis (*green arrows* in **c** and **d**)

Radiographs demonstrate reduced disc space, blurred paradiscal margins, increased paravertebral soft tissue opacity with and without calcifications, vertebral body destruction and severe kyphotic angulation in advanced progressive cases. CT is considered superior to MR imaging for visualization of small bony fragments and demonstrates more fine bony details of lytic lesions, endplate destruction and sclerotic margins, while MR imaging is considered superior for accurately defining the epidural extension of the disease and neural structure involvement. Findings of loss of vertebral body cortical definition and the presence of a calcified paraspinal mass with thick irregular rim enhancement favor tubercular over bacterial spondylodiscitis [23, 31]. Because of its superior ability to detect marrow changes before any bony destruction, MR imaging plays an important role in early diagnosis even in patients with normal radiographs. In majority of cases, tubercular spondylitis appears hyperintense on T2-weighted images and hypointense on T1-weighted images with contrast enhancement indicating marrow edema in the infected area (Fig. 8). An important imaging feature that characterizes tuberculous infection compared to bacterial infection is sparing of the intervertebral disc in the early stage of infection. Conversely, early spread to discs with loss of disc height and disc herniation favor bacterial infection [4]. Other characteristic involvement of the anterior vertebral body corner, subligamentous spread, multiple vertebral bodies, extensive paraspinal abscess formation, abscess calcification, and vertebral destruction differentiates tubercular from bacterial spondylodiscitis [17, 23, 32].

With progression of the disease classic discovertebral involvement can be seen which appear hyperintense on T2-weighted images and hypointense on T1-weighted images. In the chronic stage due to delayed diagnosis, low signal intensity on T1 and T2-weighted images indicates vertebral collapse with endplate sclerosis. During treatment, progressive increase in vertebral signal intensity on T1-weighted images suggests fatty replacement and indicates healing [23, 31, 32]. The infective process can extend into the epidural space causing cord compression, which is associated with a high mortality [23]. Extension into the spinal canal can lead to arachnoiditis (Fig. 8) and tuberculomas in the brain parenchyma in some cases.

## Imaging features of pathologies mimicking spinal infections

**Fig. 9:**
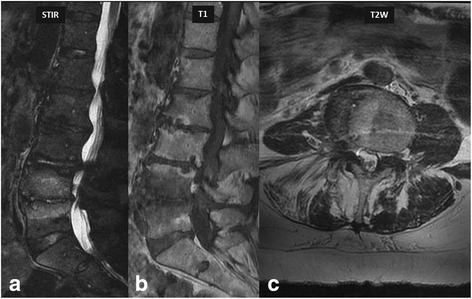
An example of the Modic changes related to degenerative disease in a 68-year-old farmer. Note the Type I Modic changes at L4–5 endplates showing hyperintense signal on STIR image (**a**) and hypointense signal on T1-weighted image (**b**). Also note the absence of any surrounding paravertebral edema or psoas involvement (**c**), differentiating these Modic changes from vertebral osteomyelitis

Many degenerative and inflammatory spinal disorders may mimic spinal infections and it is necessary to be able to differentiate them from infectious spondylitis. In patients with overlapping clinical presentations, Modic type I degenerative changes can mimic infectious spondylitis as it can have endplate bone marrow edema with areas of contrast enhancement (Fig. 9). However, lack of abnormally increased signal in the disc on T2 weighted images and lack of soft tissue involvement including epidural abscess favors a degenerative disease over an infection [7].

**Fig. 10:**
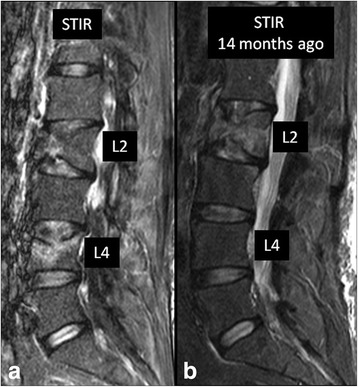
74-year-old man presenting with acute low back pain following a fall. Sagittal STIR image at the time of presentation (**a**) shows end-plate deformities and bone marrow edema involving L2 and L4 vertebral bodies. When compared to prior study from 14 months ago (**b**), it became clear compression fracture of L2 is actually chronic. Change involving L4 is a new finding consistent with acute Schmorl’s node in this patient who had no clinical or laboratory evidence of active infection

Acute traumatic schmorl’s node is the extrusion of a disc into the endplate (Fig. 10). Due to associated vascularization and inflammation, bone marrow edema and contrast enhancement may be identified, and imaging features may be indistinguishable from those of infectious spondylitis. However, the presence of a high-signal-intensity concentric ring surrounding a cartilaginous node and involvement of only one endplate without disc signal abnormality favor acute traumatic schmorl’s node over infection [8].

**Fig. 11:**
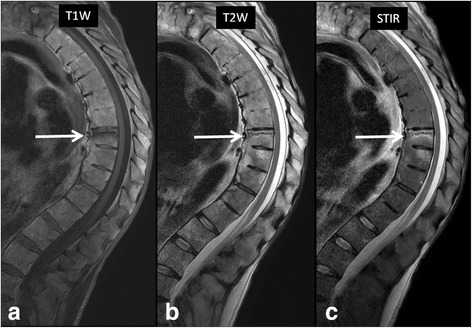
52-year-old man with known history of ankylosing spondylitis presented with acute back pain following a fall. **a** T1-weighted, **b** T2-weighted and **c** STIR images in the sagittal plane shows a horizontal fracture *line* with associated bone marrow edema involving the superior endplate of T8 vertebral body (*arrows*). The patient showed no clinical evidence of active infection

A common complication of advanced ankylosing spondylitis is spinal fractures which can be either spontaneous or following a trauma. These are commonly caused by osteoporosis in a patient with spinal fusion. These fractures typically are three-column fractures involving juxtadiscal endplate or the disk space. Pseudoarthrosis may develop at the site of the fracture resulting in endplate erosion and bone marrow edema mimicking imaging findings of infectious spondylitis (Fig. 11) [9]. A history of ankylosing spondylitis, proper clinical context and extension of the fracture line into the posterior elements help in differentiating ankylosing spondylitis related pseudoarthrosis from infectious spondylitis.

**Fig. 12:**
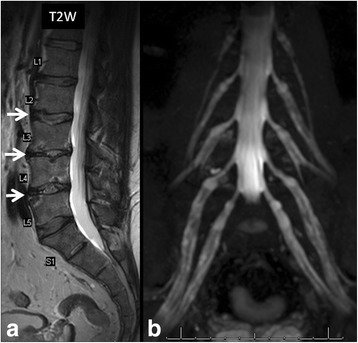
64-year-old man with diabetes mellitus with low back and bilateral leg pain consistent with diabetic neuropathy. Sagittal T2-weighted image (**a**) shows end plate fragmentation at multiple lumbar levels (L2/3, L3/4, L4/5, *arrows*). MR neurography image (**b**) shows diffuse lumbosacral plexopathy with patchy thickening of all nerves. The patient showed no clinical evidence of active infection

In patients with diabetes mellitus, syringomyelia, syphilis, or other neuropathic disorders, diminished protective sensation to repetitive trauma results in neuropathic arthropathy. In the spine, the thoracolumbar junction and the lumbar regions are most commonly affected. The clinical presentation and imaging appearance of spinal neuropathic arthropathy may resemble those of severe degenerative disease or spinal infection (Fig. 12). In the spinal neuropathic arthropathy, destructive changes in the vertebral bodies lead to a fracture, followed by bone sclerosis, new bone formation, a loss of disc space followed by pseudoarthrosis in the end stage. However, low signal in the disc and surrounding marrow on T2-weighted images are more likely seen in the neuropathic spine than in bacterial spondylitis. Other findings of vacuum phenomenon, facet involvement, osseous joint debris and joint disorganization are also suggestive of spinal neuropathic arthropathy [10].

**Fig. 13:**
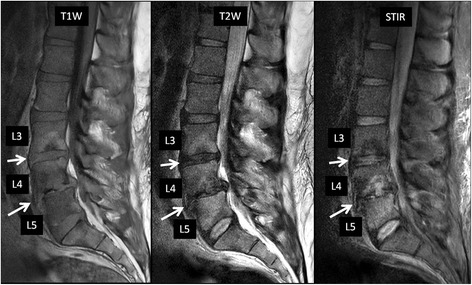
39-year-old man with chronic renal failure on dialysis, with biopsy-proven amyloid spondyloarthropathy involving the lumbar spine. There are disc space narrowing and end-plate desctruction with associated bone marrow edema at L3/4 and L4/5. There is also grade 1 anterolisthesis of L4 over L5 as well as a disc herniation causing severe central canal stenosis at L4/5. From the imaging findings, initially infectious spondylodiscitis was suspected. However, failure to respond to antibiotic therapy prompted biopsy, which was negative for infectious organism and positive for amyloid stain

Destructive spondyloarthropathies can also be seen in patients with long-term hemodialysis and resultant amyloid deposition in the spine (Fig. 13). Although MR imaging appearances can be similar to those of infection, these patients are usually clinically silent and bone marrow edema is not as prominent as infection [33]. Differentiation between infectious spondylodiscitis and amyloid spondylopathy can be achieved by biopsy, with negative culture and positive stain for amyloid.

## Conclusions

A better understanding of the clinical features and pathophysiologic basis of bacterial and tuberculous spondylodiscitis contributing to their imaging appearances help the radiologist to make an accurate diagnosis and differentiate infections from other abnormalities which may mimic infections. This may result in early diagnosis and prompt initiation of appropriate treatment to avoid complications such as abscess formation, spinal deformities and neurological deficits.

## Abbreviations

CNS: Central nervous system
CRP: C-reactive protein
CSF: Cerebrospinal fluid
CT: Computed tomography
DWI: Diffusion weighted imaging
ESR: Erythrocyte sedimentation rate
MR: Magnetic resonance
STIR: Short tau inversion recovery
T1W: T1 weighted
T2W: T2 weighted
TB: Tuberculosis
TE: Time to echo
